# Supplementary material for: Content-rich biological network constructed by mining PubMed abstracts
Source: BMC Bioinformatics. 2004 Oct 8;5:147. doi: 10.1186/1471-2105-5-147 (PMC528731; doi:10.1186/1471-2105-5-147)
Supplement: Additional File 2 — The original results of the above study (non-essential files are deleted to keep the file size under the limit set by BMC bioinformatics). [file 1471-2105-5-147-S2.bz2 › chilibotAdditionalFile2/dip40/330ID1328224E3926/html/TNF_TNFRSF1B.html]

 


 **TNF** and **TNFRSF1B** 
  
Found 687 abstracts in PubMed, retrieved 40.  
 

 What does Google say? 
 PDF only 
| .edu only 

---

**Interactive relationship** (e.g. stimulation, inhibition, etc)

**Neutral relationship**- RESULTS Both infliximab and etanercept  [ **TNFRSF1B** ]  neutralized  **TNF**  alpha  [ **TNF** ]  effectively.  Ref: 12806611 Gastroenterology, 2003
- The  **TNF**  binding moiety of etanercept  [ **TNFRSF1B** ]  is derived from soluble  **TNF**  receptor subunits.  Ref: 12747285 Clin Exp Rheumatol< MedlineTA>Clin Exp Rheumatol, 2003
- CONCLUSIONS Although both infliximab and etanercept  [ **TNFRSF1B** ]  showed powerful  **TNF**  alpha  [ **TNF** ]  neutralization, only infliximab was able to bind to PBL and lamina propria T cells and subsequently to induce apoptosis of activated lymphocytes.  Ref: 12806611 Gastroenterology, 2003
- LT consists of soluble LT alpha 3, which binds to  **TNF**  R1 and  **TNF**  R2  [ **TNFRSF1B** ] , and membrane LT alpha 1 beta 2, which binds to LT beta R.  Ref: 12882833 Int Immunol, 2003
- While etanercept  [ **TNFRSF1B** ]  is a 75kD  **TNF**  receptor fusion protein that binds to TNFalpha and TNFbeta, infliximab is a chimeric monoclonal antibody that binds to TNFalpha both in its soluble form in the serum and on the cell membrane.  Ref: 12749755 BioDrugs, 2003
- A new promising therapeutic alternative is represented by anti tumour necrosis factor alpha  **TNF**  alpha  [ **TNF** ]  drugs such as infliximab and etanercept  [ **TNFRSF1B** ] .  Ref: 12817067 Nephrol Dial Transplant, 2003
- The importance of  **TNF**  alpha  [ **TNF** ]  and apoptosis in psoriasis is shown through the review of clinical trials using anti  **TNF**  alpha  [ **TNF** ]  immunobiologics e.g. etanercept  [ **TNFRSF1B** ] , infliximab and apoptosis inducing treatments that result in clinical improvement of the disease.  Ref: 12851985 J Drugs Dermatol, 2002
- Moreover, renal  **TNF**  alpha  [ **TNF** ]  expression, ICAM 1 expression and serum  **TNF**  alpha  [ **TNF** ]  levels were lower in TNFR2  [ **TNFRSF1B** ]  deficient mice compared with wild type or TNFR1 deficient mice treated with cisplatin.  Ref: 12865254 Am J Physiol Renal PhysiolAm J Physiol Renal Physiol, 2002
- Etanercept  [ **TNFRSF1B** ]  is a dimeric fusion protein that binds specifically to  **TNF**  alpha  [ **TNF** ] , rendering it biologically inactive.  Ref: 12853521 Chest, 2003
- Both  **TNF**  receptors were detected, with  **TNF**  R1 being prominent on bronchial epithelial cells and endothelial cells, and  **TNF**  R2  [ **TNFRSF1B** ]  being expressed by nearly all cell types.  Ref: 12849708 Cytokine, 2003
- Inactivation of  **TNF**  alpha  [ **TNF** ]  and IL 1beta was achieved by repetitive intraperitoneal injection of etanercept  [ **TNFRSF1B** ]  and anakinra, respectively.  Ref: 12883478 HepatologyHepatology, 2003
- These results indicate that TNFR2  [ **TNFRSF1B** ]  participates in cisplatin induced renal injury in mice and may play an important role in  **TNF**  alpha  [ **TNF** ]  mediated inflammation in the kidney in response to cisplatin.  Ref: 12865254 Am J Physiol Renal PhysiolAm J Physiol Renal Physiol, 2003
- Neither absolute levels of  **TNF**  alpha  [ **TNF** ]  nor  **TNF**  alpha  [ **TNF** ]  activity in the serum, BAL fluid, or alveolar macrophages were able to predict which patients would respond to etanercept  [ **TNFRSF1B** ] .  Ref: 12853521 Chest, 2003
- In addition, a small number of opportunistic infections have been observed with the  **TNF**  inhibitor, etanercept  [ **TNFRSF1B** ] .  Ref: 12787529 Best Pract Res Clin Rheumatol, 2003
- Of the agents that are currently approved by the US Food and Drug Administration FDA for this indication, etanercept  [ **TNFRSF1B** ]  and infliximab neutralize tumor necrosis factor  [ **TNF** ]   **TNF** , and anakinra inhibits interleukin 1 IL 1.  Ref: 12747285 Clin Exp Rheumatol< MedlineTA>Clin Exp Rheumatol, 2003

**Non-interactive relationship** (e.g. studied together, co-existance, homology, etc.)

- METHODS We used a nuclear factor kappaB reporter assay and a cytotoxicity bioassay to study  **TNF**  alpha  [ **TNF** ]  neutralization by infliximab and etanercept  [ **TNFRSF1B** ] .  Ref: 12806611 Gastroenterology, 2003
- The serum concentrations of  **TNF**  alpha  [ **TNF** ] , soluble  **TNF**  receptor 1 sTNF R1,  **TNF**  R2  [ **TNFRSF1B** ] , IL 4, IL 5, IL 8, ECP, and IgE were studied in symptom free adults n = 22 and their children n = 22 with bronchial hyperreactivity.  Ref: 12776444 Allergy Asthma Proc< MedlineTA>Allergy Asthma Proc, 2003
- Etanercept  [ **TNFRSF1B** ] , a recombinant  **TNF**  receptor immunoglobulin G fusion protein, is highly effective in rheumatoid arthritis but not in Crohn s disease.  Ref: 12806611 Gastroenterology, 2003
- The difference in  **TNF**  R II  [ **TNFRSF1B** ]  levels between seropositives and AIDS was however more impressive.  Ref: 12815299 Methods Cell Sci, 2002
- OBJECTIVE To study the distribution of clinical responses to treatment with the tumor necrosis factor alpha  [ **TNF** ]  TNFalpha antagonists etanercept  [ **TNFRSF1B** ]  and infliximab, and in particular, to determine whether there is a biologically meaningful distinction between responders and nonresponders.  Ref: 12794816 Arthritis Rheum, 2003
- It turned out that antibodies to  **TNF**  R1, but not to  **TNF**  R2  [ **TNFRSF1B** ]  reduced hyperalgesia, indicating that  **TNF**  R1 is the receptor concerned.  Ref: 12799919 Schmerz, 1999
- Therefore, we measured Tnfr2  [ **TNFRSF1B** ]  mRNA in kidney, adrenal, heart, and aorta from rats with ACTH induced, corticosterone induced, and spontaneous HT SHR, and tested the effect of blockade of  **TNF**  alpha  [ **TNF** ]  by a recombinant  **TNF**  R2  [ **TNFRSF1B** ]  fragment huTNFR Fc on development of HT in the ACTH model.  Ref: 12878376 Am J Hypertens, 2003
- Two candidate genes for bone mineral density BMD, tumor necrosis factor alpha  [ **TNF** ]  receptor 2  **TNFRSF1B**  and lysyl hydroxylase PLOD1, have been scanned for single nucleotide polymorphisms SNPs within their coding and promoter regions.  Ref: 12754673 Calcif Tissue Int< MedlineTA>Calcif Tissue Int, 2003
- Additionally, fusion constructs of the transmembrane domain of LMP1 and the intracellular signaling domain of CD40,  **TNF**  R1, and  **TNF**  R2  [ **TNFRSF1B** ]  likewise transactivated the CD83 promoter via NF kappaB.  Ref: 12857898 J VirolJ Virol, 2003
- We report three consecutive patients with critical chronic refractory ITP, who responded promptly and completely following treatment with etanercept  [ **TNFRSF1B** ] , an inhibitor of tumor necrosis factor alpha  [ **TNF** ] .  Ref: 12749017 Am J Hematol, 2003
- TCR activation in NF kappaB deficient Do 11.10 cells resulted predominantly in tumor necrosis factor  [ **TNF** ]  receptor 2 TNFR2  [ **TNFRSF1B** ]  dependent bystander cell death rather than classical Fas dependent AICD.  Ref: 12813034 J Biol Chem< MedlineTA>J Biol Chem, 2003
- 252 G A, and TNFR2  [ **TNFRSF1B** ]  196 R M in 94 Korean BD patients and age and sex matched healthy controls to investigate the role of  **TNF**  and  **TNF**  receptor polymorphisms in BD.  Ref: 12770792 Hum Immunol< MedlineTA>Hum Immunol, 2003
- Two closely related variants, TNFalpha and TNFbeta, elicit various cellular responses via two distinct  **TNF**  receptors, the 55 kDa  **TNF**  R1 and the 75 kDa  **TNF**  R2  [ **TNFRSF1B** ] .  Ref: 12849708 Cytokine, 2003
- As  **TNF**  exerts its effects via two receptors,  **TNF**  receptor 1  **TNF**  R1 and  **TNF**  receptor 2  **TNF**  R2  [ **TNFRSF1B** ] , we also investigated, which of the receptors is relevant to the causation of pain in this model.  Ref: 12799919 Schmerz, 1999
- The upregulation of TNFR2  [ **TNFRSF1B** ]  but not TNFR1 was blunted in  **TNF**  alpha  [ **TNF** ]  deficient mice indicating ligand dependent upregulation of TNFR2  [ **TNFRSF1B** ] .  Ref: 12865254 Am J Physiol Renal PhysiolAm J Physiol Renal Physiol, 1999
- OBJECTIVE A recent Italian study found that homozygosity for the G allele of the 196 single nucleotide polymorphism SNP of the tumor necrosis factor  [ **TNF** ]  receptor II  **TNFRSF1B**  gene was more prevalent in patients with severe rheumatoid arthritis RA.  Ref: 12858434 J Rheumatol, 2003
- This activity of LT depends on membrane LT alpha 1 beta 2 and not soluble LT alpha 3, since DA virus persisted and induced inflammatory demyelination in LT beta R, but not  **TNF**  R1 or  **TNF**  R2  [ **TNFRSF1B** ] , mice.  Ref: 12882833 Int Immunol, 2003
- In human hypertension HT plasma tumor necrosis factor  [ **TNF** ]   **TNF**  alpha  [ **TNF** ]  and soluble  **TNF**  receptor 2 fragment sTNF R2 are increased, and the  **TNF**  R2  [ **TNFRSF1B** ]  gene  **TNFRSF1B**  has been implicated.  Ref: 12878376 Am J Hypertens, 2003
- It is concluded that a combination of absolute number of CD4 cells and  **TNF**  R II  [ **TNFRSF1B** ]  assay along with clinical evaluation may be used to monitor therapy in resource poor countries where frequent viral load assay is unaffordable.  Ref: 12815299 Methods Cell Sci, 2002
- We explored the possible involvement of these cytokines during the main phase of lymphocyte recruitment into the axotomised facial motor nucleus 7 21 days after nerve cut using mice homozygously deficient for IL1 receptor type 1 IL1R1,  **TNF**  receptor type 1 TNFR1, type 2 TNFR2  [ **TNFRSF1B** ]  and type 1 and 2 TNFR1& ; 2, IFNgamma receptor type 1 IFNgammaR1, and the appropriate controls for the genetic background.  Ref: 12774313 J Neurosci Res, 2003
- BACKGROUND Etanercept  [ **TNFRSF1B** ] , a recombinant biologic anti tumor necrosis factor  [ **TNF** ]   **TNF**  alpha  [ **TNF** ]  therapeutic, is approved for the treatment of certain autoimmune arthritides by subcutaneous SC injection.  Ref: 12809967 Clin Ther, 2003
- Rare cases of IgA glomerulonephritis during immunomodulating therapy with leflunomide and  **TNF**  blocking receptor etanercept  [ **TNFRSF1B** ]  are reported.  Ref: 12874640 Reumatismo, 2003
- Because both infliximab and etanercept  [ **TNFRSF1B** ]  are  **TNF**  alpha  [ **TNF** ]  neutralizing drugs, we investigated the differences in  **TNF**  alpha  [ **TNF** ]  neutralizing capacity and human lymphocyte binding and apoptosis inducing capacity of both molecules.  Ref: 12806611 Gastroenterology, 2003
- In situ localization of TNFalpha beta, TACE AND  **TNF**  receptors  **TNF**  R1 and  **TNF**  R2  [ **TNFRSF1B** ]  in control and LPS treated lung tissue.  Ref: 12849708 Cytokine, 2003
- By examining surface expression of numerous candidate molecules tumor necrosis factor  [ **TNF** ]  receptor TNFR 1, TNFR2  [ **TNFRSF1B** ] , CD36, CD40, CD47, CD80, CD86, PD L1, CD95 ligand, and type I interferon receptor, we report that IPE cells uniquely express on their surface the costimulatory molecule CD86.  Ref: 12835481 J Exp Med, 2003
- OBJECTIVE To analyse whether polymorphisms of several cytokine genes are associated with the responsiveness to  **TNF**  blockade with etanercept  [ **TNFRSF1B** ] .  Ref: 12759288 Ann Rheum Dis< MedlineTA>Ann Rheum Dis, 2003
- Etanercept  [ **TNFRSF1B** ] , a competitive inhibitor of  **TNF**  alpha  [ **TNF** ] , is currently FDA approved for psoriatic arthritis and rheumatoid arthritis.  Ref: 12894133 J Am Acad Dermatol, 2003
- Associations of HLA DRB1, tumor necrosis factor  [ **TNF** ]  a promoter  **TNF** ,  **TNF**  receptor 2 TNFR2  [ **TNFRSF1B** ] , Fcg receptor IIa FCGR2A, IIb FCGR2B, IIIa FCGR3A, IIIb FCGR3B, and CTLA 4 CTLA4 polymorphisms were examined in a case control analysis.  Ref: 12858454 J Rheumatol, 2003
